# *In Vitro* Antimicrobial and Cytotoxic Effects of Solvent-Fractionated Extracts from *Raphionacme hirsuta* (E.Mey.) R.A.Dyer (Apocynaceae) Bulbs

**DOI:** 10.3390/life16071154

**Published:** 2026-07-13

**Authors:** Nkoana I. Mongalo, Maropeng V. Raletsena, Nontokozo Magwaza, Perpetua Modjadji

**Affiliations:** 1 College of Agriculture and Environmental Sciences Laboratories, University of South Africa, Private Bag X6, Florida 1710, South Africa; 2 Institute for Nanotechnology and Water Sustainability, College of Science, Engineering and Technology, University of South Africa, Johannesburg 1710, South Africa; 3 Department of Public Health, School of Health Care Sciences, Sefako Makgatho Health Sciences University, 1 Molotlegi Street, Ga-Rankuwa 0208, South Africa; 4 Non-Communicable Diseases Research Unit, South African Medical Research Council, Tygerberg, Cape Town 7505, South Africa; 5 Department of Life and Consumer Sciences, College of Agriculture and Environmental Sciences, University of South Africa, Florida Campus, Johannesburg 1709, South Africa

**Keywords:** ethnomedicine, *Raphionacme hirsuta*, antimicrobial, anticancer, antineoplastic, GC-TOF-MS

## Abstract

Antimicrobial resistance in various microorganisms and opportunistic pathogens associated with HIV/AIDS poses a serious threat to human life and healthcare systems worldwide. Different forms of cancer are likely to arise in immunocompromised patients. The antimicrobial and anticancer effects of methanol extract and fractions from *Raphionacme hirsuta* have been investigated. The carbon tetrachloride fraction showed a remarkably low minimum inhibitory concentration (MIC) of 0.02 mg/mL against *Escherichia coli*, *Mycoplasma hominis*, and *Cryptococcus neoformans*, while the *n*-hexane fraction showed a similar MIC against *C. neoformans*. Furthermore, the carbon tetrachloride fraction exhibited promising IC_50_ values of 18.21 and 25.22 µg/mL against HeLa and MCF-7 cancerous cell lines, respectively. The fraction was subjected to GC-TOF-MS and yielded four major compounds, including 7,9-Di-tert-butyl-1-oxaspiro (4,5) deca-6,9-diene-2,8-dione (5.322%), Hexanedial (3.691%), 4-(4-tert-Butylphenyl)-1,3-thiazol-2-ylamine (3.329%), and Di-n-decylsulfone (3.201%). These substances could potentially account for the plant species’ initial biological activity, which is why it is necessary to investigate their in vivo actions. The gummy extract had less biological activity than the fractions. To the best of our knowledge, this is the first study to report the antimicrobial and anticancer activities, as well as the phytochemistry, of the plant species.

## 1. Introduction

The global use of antibiotics in the treatment and management of human and animal infections is a crucial and integral part of healthcare systems worldwide. However, some important pathogens have recently developed some resistance to such antibiotics, particularly those used in developing countries [1]. Indeed, antimicrobial resistance is a recognized global problem that is further exacerbated by alarming new HIV-AIDS infections directly related to tuberculosis (TB) infections, misuse of antibiotics, misdiagnosis of various pathogenic infections, and early-stage diseases, infection, and other social factors [2]. The most common mechanism of antimicrobial resistance involves enzymatic degradation, target alteration, reduced uptake, and overexpression of efflux pump proteins [3]. However, it is important to note that most rural communities rely on medicinal plants as a first line of prevention and treatment for multiple pathogenic infections [4,5]. The global use of antibiotics to treat infections in humans and animals is an important part of healthcare systems worldwide. However, some important pathogens have recently developed some resistance to such antibiotics, particularly those used in developing countries. Indeed, antimicrobial resistance is a recognized global problem, further compounded by alarming new HIV-AIDS infections directly related to TB infections, misuse of antibiotics, misdiagnosis of various pathogenic infections and diseases in the early stages of infection, and other social factors [6,7]. The most common mechanism of antimicrobial resistance involves enzymatic degradation, target alteration, reduced uptake, and overexpression of efflux pump proteins [4]. However, it is important to note that most rural communities rely on medicinal plants as the first line of prevention and treatment of multiple pathogenic infections [4,5].

There are 37 species in the genus *Raphionacme* Harv., which are primarily geophytic herbaceous plants. Most of these species are erect, with a few being climbers or prostrate palms [8,9]. The species is found all over Africa, but it is most prevalent in the southern African savannah and tropical grassland biomes [10]. *Raphionacme hirsuta*, commonly called “Tshengwa”, is a tiny, branched perennial plant, 50–200 mm tall, with herbaceous, finely hairy stems containing milky sap and a broad, tuberous base. Its flowers are star-like and vary in colour, ranging from white, purple, and light blue. The whole plant is reportedly used in the treatment of a plethora of opportunistic infections associated with tuberculosis, skin problems, various forms of cancers, and sexually transmitted diseases that include syphilis and gonorrhea [11,12,13,14]. Bulbs may be cut into pieces and immersed in hot water for a few days to make an alcoholic beverage which may be drunk by older men, “kgorong,” or in ancestral ceremonies. The current work is aimed at investigating the antimicrobial efficacy and cytotoxicity of *R. hirsuta* and its fractions. Furthermore, to explore the phytochemistry of the biologically active fraction using gas chromatography time of flight mass-spectrometry (GC-TOF-MS).

## 2. Materials and Methods

### 2.1. Identification, Extraction, and Fractionation of Plant Material

**Figure 1:**
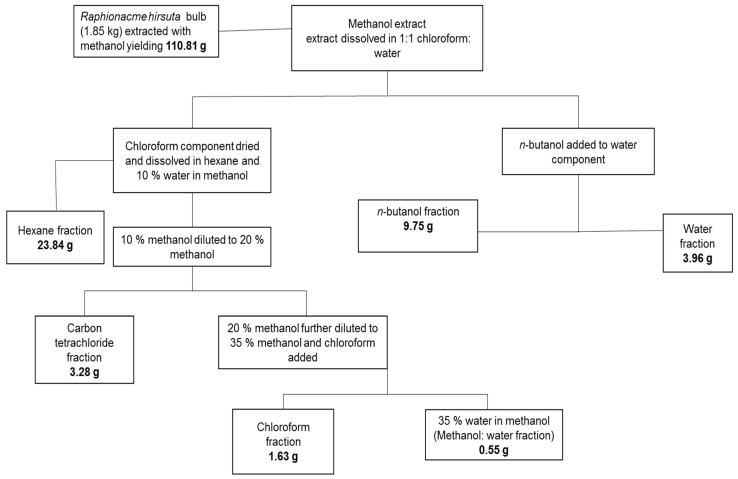
Schematic presentation of the solvent–solvent fractionation of *Raphionacme hirsuta* methanol extracts bulbs.

*Raphionacme hirsuta* bulbs were collected in June 2022 from a bush field near Phala High School in Senwabarwana, Blouberg District, Limpopo Province (Latitude: −23°08′21.12″ S and Longitude: 29°00′4.68″ E), with assistance from a traditional healer, Mr. Benjamin Mokgehle. Voucher specimens were provided for identification, which the National Botanical Institute in Pretoria conducted, and a specimen has been deposited in the herbarium of the University of South Africa in Florida Park, Johannesburg (MNI-33). Bulbs were cut into smaller pieces and dried in an oven at 35 °C. The dried plant materials were ground into a fine powder using a Scientec Hammer Mill (Hamburg, Germany) and extracted with methanol (AR grade) in a 1:5 *w*/*v* ratio using methanol as an extracting solvent. Methanol extract from *Raphionacme hirsuta* bulbs was further subjected to solvent–solvent fractionation using a separatory funnel, as shown in Figure 1 below [15]. The methanol extract was briefly dissolved in 600 mL of 1:1 chloroform: distilled water and separated. The aqueous fraction was mixed with *n*-hexane to give aqueous and hexane fractions. Water was evaporated using the instrument SP Genevac EZ-2 4.0 Elite (Qiagen N.V., Frankfurt, Germany) at 65 °C overnight. The chloroform was evaporated using a rotary evaporator and dissolved in 1:1 hexane:10% methanol, which was also separated to give a hexane fraction, and the aqueous methanol was further diluted to 20% water in methanol and then equally divided to give carbon tetrachloride, and the aqueous methanol, which was further diluted to 35% water in methanol. Chloroform was added to a 35% aqueous methanol solution and separated using a funnel to yield a chloroform fraction and methanol–water fractions. In all cases, volumes of solvents used were evaporated using EZ-2. (Frankfurt, Germany). All the reagents used for extraction and fractionation were obtained from Sima Aldrich, Darmstadt (Germany) and were of AR Grade.

### 2.2. Antimicrobial Activity

#### 2.2.1. Selection of Microorganisms

Eight pathogenic microorganisms, such as *Candida albicans* (ATCC 24433), *Cryptococcus neoformans* (clinical isolate), *Escherichia coli* (ATCC 25922), *Moraxella catarrhalis* (Clinical isolate), *Klebsiella pneumoniae* (13883), *Mycoplasma hominis* (ATCC 15488), *Bacillus cereus* (ATCC 10702), and *Staphylococcus aureus* (6538), were selected in the current study. *C. neoformans* and *M. catarrhalis* were isolated from two HIV-AIDS patients with wounds on the legs and excessive cough, respectively. The microbes were selected based on the documented medicinal plant use from the literature. All the growth media were obtained from Sigma Aldrich (Schnelldorf, Germany).

#### 2.2.2. Bioautography of Fractions from *Raphionacme hirsuta*

The method of Begue and Kline [16] was adopted for carrying out bioautography of the extract and fractions against *Staphylococcus aureus*, *Klebsiella pneumoniae*, *Bacillus cereus*, and *Candida albicans*. The extract did not yield any inhibition, hence excluded. Each of the fractions, about 30 µL of 10 mg/mL stock solution dissolved in methanol, was separately spotted in the form of a band on the bottom (2 cm) of a silica gel-coated TLC plate and run with BEA (benzene 90 mL: ethanol 10 mL: ammonium hydroxide 1 mL) as mobile phase. This mobile phase was selected because it separated compounds better than other mobile phases, such as EMW (ethyl acetate 5 mL: methanol 5.4 mL: water 4.4 mL) and CEF (Chloroform 5 mL: ethyl acetate 4 mL: formic acid 1 mL). The plates in the TLC tank were monitored, and a pencil was used to mark the solvent front. The plates were allowed to dry in a laminar flow cabinet for 3 days, then sprayed with a confluent growth of microorganisms of choice and incubated overnight at 37 °C in a moist environment. The next day, plates were sprayed with 2 mg/mL of *p*-iodonitro-tetrazolium chloride (INT, Sigma Aldrich, Schnelldorf, Germany) and then re-incubated until a pinkish colour appeared on the plates. Clear spots indicating activity of the fractions and patterns of antimicrobial compounds were marked and compared.

#### 2.2.3. Antibacterial Activity of Methanol Extract and Fractions Using Microdilution Assay

The selected extract and fractions’ minimal inhibitory concentrations (MICs) were tested using the broth microdilution assay [17]. An overnight culture of each microorganism was diluted in fresh Nutrient broth (Oxoid) to a concentration of approximately 1.1 × 10^7^ CFU/mL. *Mycoplasma hominis* was grown on Mycoplasma agar (Merck, Modderfontein, South Africa) supplemented with mycoplasma supplement G. *M. hominis* was maintained on Mycoplasma agar, while other bacterial strains were maintained on Nutrient agar (Bioculture, Anatech, South Africa). A volume of 100 µL of extract compounds and fractions (50 mg/mL in 5% DMSO) was added to a multi-well plate containing 100 µL of sterile distilled water and was two-fold serially diluted. Then, 100 µL of bacterial culture was added to each well. Vancomycin, amphotericin B, and streptomycin served as positive controls. The plates were incubated overnight at 37 °C, except for *M. hominis* plates, which were incubated for 24 h. Approximately 40 µL of 0.2 mg/mL freshly prepared *p*-iodo-nitro-tetrazolium chloride (INT) was added to each well and incubated for 30 min at the same temperature. The MIC was determined as the lowest concentration of the extract that inhibited bacterial growth.

#### 2.2.4. Antifungal Activity of Methanol Extract and Fractions Using Microdilution Assay

Masoko et al. [18] approach was used to evaluate the antifungal activity of the extract and its fractions against *Candida albicans* and *Cryptococcus neoformans*. In short, a 20 mg/mL stock solution of plant materials was diluted serially, and a similar concentration of the fungal strains as in 3.3 was added. Afterwards, approximately 0.02 mg/mL of freshly produced INT was cultured overnight. Following a 48 h incubation period, the data were read. In both antibacterial and antifungal activity, the final concentration of DMSO was ≈1% and did not affect the growth of the selected microorganisms. Each of the experiments was trialled three times, independently.

### 2.3. Cytotoxic Effects of Extract and Fractions from R. hirsuta

The cytotoxic effects of the extract and its fractions were evaluated using the tetrazolium-based colorimetric (MTT) assay, as described by Mosman [19]. Human breast adenocarcinoma (MCF-7) cells, human cervical cancer cells (HeLa), and human colorectal carcinoma cells (Caco-2) were all cultured to confluency in 75 cm^3^ flasks. After trypsinization, the cells were plated into 96-well plates at a seeding density of 23 × 10^4^ cells per well and incubated overnight at 37 °C with 5% CO_2_. The medium was then removed and replaced with fresh media (MEM + Glutamax + antibiotics + 10% fetal bovine serum). Subsequently, extracts and fractions dissolved in Dimethyl sulfoxide (DMSO) were added, and the plates were incubated for 48 h. After incubation, the test samples were removed from the wells, and 200 µL of fresh medium was added along with 30 µL of 5 mg/mL MTT (Melford Biolaboratories, Ipswich, South Africa). The plates were then incubated for an additional 4 h. Following MTT incubation, the media in each well were removed, and 50 µL of DMSO was added to each well to dissolve the formed formazan crystals. The plates were gently shaken to dissolve the crystals, and the absorbance was measured at 570 nm to assess MTT reduction. The percentage of cell viability was calculated using the following formula [20]:

% Cell viability = Mean absorbance of sample/Mean absorbance of control × 100.

The concentration of the test sample that caused a 50% decrease in absorbance when compared to untreated cells was used to compute the IC_50_ values (lethal concentration at which 50% of the cells are killed) from linear regression graphs. The quantity of live cells present directly correlates with the intensity of the MTT formazan generated by metabolically active live cells. Each of the experiments was carried out three times independently. DMSO was used as a negative control and did not inhibit the growth of the selected cell lines *in vitro*. The extracts were tested in a range concentration of 0 to 1000 µg/mL. The cells used in the study were obtained from Sigma Aldrich, Frankfurt, Germany.

### 2.4. GC-TOF-MS Analysis of Carbon Tetrachloride Fraction

The carbon tetrachloride fraction exhibited potent antimicrobial and anticancer activity, hence selected for phytochemical analysis using gas chromatography mass spectrometry (GC-TOF-MS) analysis. The analysis was performed using the method used in our previous studies with minor modifications [21,22]. A smaller amount of about 0.01 mg of the extract was completely dissolved in acetonitrile (GC-MS grade, Sigma Aldrich, Schnelldorf, Germany). Separation of phytocompounds was performed using gas chromatography (8890 GC-TOF-MS, Agilent Technologies, Santa Clara, CA, USA) equipped with a LECO Pegasus BT 4D Flight Mass Spectrometry Time (TOF-MS) obtained from LECO Corporation, St. Joseph, MI, in the United States of America. Shortly thereafter, the prepared sample was loaded into the preheated Gerstel MPS2 Liquid/HS/SPME autosampler (Gerstel, Mülheim an der Ruhr, Germany). The AJ & W capillary column Rxi-5Sil MS 30 × 0.25 mm I.D. (Restek, Bellefonte, PA, USA) with a film thickness of about 0.25 µM was used for the chromatographic separation. The conditioning in the chromatographic separation involved 1 µL of the sample at 1 mg/mL injected at 250 °C with a splitless injector, and the GC oven was programmed at 70 °C for 0.5 min, then ramped at 10 °C per minute to 150 °C and held for 2 min, followed by a ramp of 10 °C per minute to 330 °C and held for 3 min, resulting in a total run time of 31.5 min. Helium, purchased from Afrox (Johannesburg, South Africa) at 99.99% purity, was used as the carrier at a constant flow of 1 mL/min until the separation was complete. The GC-TOF-MS interface temperature was set at 250 °C, and the mass spectra were obtained in full scan mode at 70 eV (*m*/*z* scan varying from 50 to 600). The collection of data was obtained using ChromaTOF 5.55.29.01.1187, which encodes the NIST MS 2.4 with 95 library, enabling compound matches.

## 3. Results

### 3.1. Antimicrobial Activity

**Figure 2:**
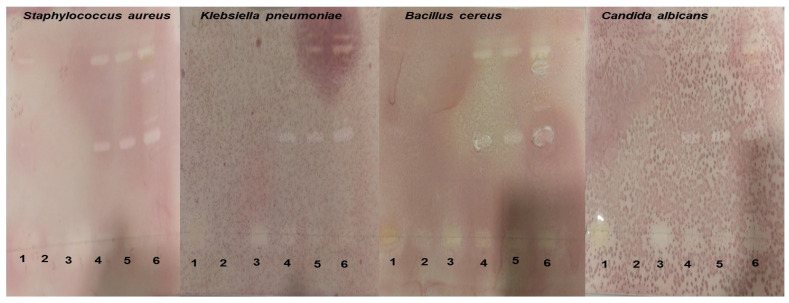
Chromatograms of fractions from *Raphionacme hirsuta* run in BEA and against several pathogens. Key: 1, Aqueous; 2, *n*-butanol; 3, *n*-hexane; 4, methanol–water; 5, chloroform; 6, carbon tetrachloride fractions.

The antimicrobial activity of the methanol extract from *Raphionacme hirsuta* and the fractions was tested for antimicrobial activity against a wide variety of microbes. In the bioautography study, fractions exhibited activity, particularly the Carbon tetrachloride fraction, yielding five compounds inhibiting the growth of *Bacillus cereus*, at a concentration of 10 mg/mL (Figure 2). Such compounds yielded R_f_ values of 0, 0.57, 0.69, 0.81, 0.86, and 0.91, with 0.69, 0.81, and 0.86 yielding a zone of inhibition. In the microdilution assay, the carbon tetrachloride fraction exhibited the lowest minimum inhibitory concentration (MIC) value of 0.02 mg/mL against *Cryptococcus neoformans*, *Escherichia coli*, and *Mycoplasma hominis*, while the *n*-hexane fraction exhibited a similar MIC value against *C. neoformans* (Table 1). The methanol extract exhibited only a notable MIC of 0.08 mg/mL against *M. hominis*. Furthermore, the extract exhibited an MIC value of 0.16 mg/mL against *B. cereus* and *Staphylococcus aureus*. The *n*-butanol fraction exhibited MICs of 0.08 and 0.04 mg/mL against *E. coli* and *M. hominis*, respectively, whereas the aqueous fraction yielded MICs ranging from 0.16 to 6.25 mg/mL. The chloroform fraction exhibited MIC values of 0.08 mg/mL against both *M. hominis* and *E. coli. M. hominis* was more susceptible to both the extract and the fractions, while *Candida albicans* exhibited lower susceptibility under the tested conditions.

### 3.2. Cytotoxic Effects of Raphionacme hirsuta Methanol Extracts and Its Fractions

The cytotoxic effects of the extract and fractions from *R. hirsuta* were studied against three different cancerous cell lines, such as Human breast adenocarcinoma (MCF-7) cells, human cervical cancer cells (HeLa), and human colorectal carcinoma cells (Caco-2) *in vitro*. (Table 2). The extract exhibited an IC_50_ value of 540.6 µg/mL against HeLa cells and a further >1000 µg/mL against both MCF-7 and Caco-2. Carbon tetrachloride fraction significantly exhibited a noteworthy anticancer effect against HeLa cells, yielding an IC_50_ value of 18.21 µg/mL. Furthermore, the extract exhibited IC_50_ values of 25.22 and 26.33 µg/mL against MCF-7 and Caco-2 cell lines, respectively, depicting a wide spectrum of cytotoxic effects.

The extract, *n*-hexane, and *n*-butanol fractions did not show any inhibition of Caco-2, yielding an IC_50_ value of >1000 µg/mL. The methanol–water fraction exhibited an IC_50_ value of 86.44 µg/mL against the MCF-7 cell line, while the chloroform fraction exhibited an IC_50_ value of 88.23 µg/mL against a similar cell line and a further IC_50_ value of 68.44 µg/mL against HeLa cells.

### 3.3. GC-TOF-MS Analysis of the Fraction

The carbon tetrachloride fraction showed strong antimicrobial and anticancer activity, so it was selected for GC-TOF-MS analysis to identify the compounds responsible for these effects. Four tentative major compounds—7,9-Di-tert-butyl-1-oxaspiro (4,5) deca-6,9-diene-2,8-dione (5.322%), hexanedial (3.691%), 4-(4-tert-butylphenyl)-1,3-thiazol-2-ylamine (3.329%), and di-n-decylsulfone (3.201%) were detected, with higher percentage areas compared to other compounds. The mass spectrometry data is attached as a Supplementary Materials.

The fraction contained a total of 47 different compounds (Table 3), including phenols, saturated fatty acid, amides, alkane hydrocarbons, flavonoids (7,9-Ditert-butyl-1-oxaspiro (4.5) deca-6,9-diene-2,8-dione), ethers (Tetrahydrofuran), and others. The similarity threshold used in the current work ranges between 500 and 950 as per the manufacturer’s specifications.

## 4. Discussion

In recent years, pathogenic microorganisms have tended to develop resistance against antibiotics used in the treatment of a plethora of infections worldwide [23]. This prompted scientists to seek traditional remedies that could serve as complementary and alternative medicines. The antibacterial activity of the extract and fractions from *R. hirsuta* bulb was evaluated using both bioautography and microdilution assay. All the extracts had a prevalent inhibition of *Candida albicans*, *Bacillus cereus*, *Klebsiella pneumoniae*, and *Staphylococcus aureus*, with R_f_ values of 0.57 and 0.86 (Figure 2). These two compounds showed a wide spectrum against the selected microbes. These suggest that the bioactive compounds from the plant species are non-polar. A similar trend has been observed elsewhere [24,25]. However, most of the fractions also had a common inhibition against the selected microbes at R_f_ value 0. This may well suggest that these compounds were not non-polar in nature, hence remained at the origin. According to Kuete et al. [26], the mode of action of many non-polar extracts and fractions may be through inhibiting the growth of *C. albicans* and some bacterial strains is mainly via inhibition of lanosterol14-α-demethylase, 3-β-D-glucansynthase, DNA/RNA synthesis, squalene-epoxidase, and binding of ergosterol. In the microdilution assay, the carbon tetrachloride fraction exhibited the lowest minimum inhibitory concentration (MIC) value of 0.02 mg/mL against *Cryptococcus neoformans*, *Escherichia coli*, and *Mycoplasma hominis*, while the extract exhibited MIC values of 0.31 and ≥5 mg/mL against similar strains, respectively (Table 2). These may well suggest that solvent–solvent fractionation significantly increased the antimicrobial efficacy of the medicinal plant extract. These findings corroborate the findings from other studies [27,28,29]. Although the fractionation increased the antimicrobial efficacy, such activities were incomparable to the control drugs used in the study. It is crucial to remember that, despite extensive research on the antimicrobial efficacy of different plant species, fractions, and isolated compounds, authors have different standards for these activities [11]. In the present study, plant extracts with MIC values of less than or equal to 0.1 mg/mL are considered active and have potential and remarkable antimicrobial action [30]. As a result, a MIC > 1 mg/mL is considered moderately active or inactive. Judging by this standard, at least two fractions had remarkable activity against *C. neoformans*, *E. coli*, and *M. hominis*, while the extract only had activity against *M. hominis*, yielding an MIC value of 0.08 mg/mL. Furthermore, *M. hominis* was the most susceptible microbe to the extract and fractions. Contrarily, other authors report the microbe to be more resistant to both plant extracts and many antibiotics, citing its morphology as a reason [31,32]. It is important to note that the bacterium lacks a cell wall around its cell membrane, which makes it naturally resistant to antibiotics such as beta-lactams, which target cell wall synthesis [33].

Worldwide, cancer infections are rapidly rising, and it is estimated that almost 1 million people per year may die by 2030, with a higher mortality rate in sub-Saharan Africa [34]. The cytotoxicity of the extract and fractions from *Raphionacme hirsuta* bulb has been evaluated against human breast adenocarcinoma (MCF-7), human cervical cancer (HeLa), and human colorectal carcinoma cells (Caco-2) *in vitro* (Table 2). The carbon tetrachloride fraction exhibited a remarkable IC_50_ value of 18.21, 25.22, and 26.22 µg/mL against HeLa, MCF-7, and Caco-2 cells, respectively, while the extract yielded an IC_50_ value of 540.6 µg/mL against HeLa cells and a further >1000 µg/mL against both MCF-7 and Caco-2 cells. These results suggest that the fractions possess a better inhibition of cancerous cell lines compared to the extract. Other authors reported a similar trend and further suggested that fractionation reduces a plethora of impurities from medicinal plant extracts [35,36]. According to the United States Cancer Institute, a plant extract is referred to as inhibitory to cancer cell lines if it exhibits an IC_50_ value of less than 30 µg/mL, while the extracts with IC_50_ values ranging from >30 µg/mL to 45 µg/mL have a moderate effect [37,38]. Judging by these standards, the carbon tetrachloride fraction exhibited potent cytotoxic activity compared to the other fractions and the extract. However, there is still a need to explore the mode of action of these fractions. Furthermore, there is a need to explore the cytotoxicity of the fraction against the normal cell line to ascertain its safety. The exudates (whitish sap) from the plant species also need to be tested for cytotoxicity against these cell lines. Although these preliminary results may validate the use of the plant species in the treatment of various forms of tumours, it is essential to isolate and characterize the possible active compounds from the plant species.

Four major compounds, such as 7,9-Di-tert-butyl-1-oxaspiro (4,5) deca-6,9-diene-2,8-dione (5.322%), hexanedial (3.691%), 4-(4-tert-butylphenyl)-1,3-thiazol-2-ylamine (3.329%), and di-n-decylsulfone (3.201%) were detected, with a higher percentage area compared to other compounds (Table 3). These compounds may the responsible for the biological activities observed. The compound 9-Di-tert-butyl-1-oxaspiro (4,5) deca-6,9-diene-2,8-dione is abundant in the plant kingdom [39,40,41]. Earlier, 9-Di-tert-butyl-1-oxaspiro (4,5) deca-6,9-diene-2,8-dione has been detected from the *Stretomycetes* species, using GC-MS analysis, and reported a potent antimicrobial activity against Methicillin-resistant *Staphylococcus aureus* (MRSA), *in vitro* [42]. Furthermore, the compound showed the highest binding affinity when docked against target proteins Fem A, MurB, MurE, and PBP2a, which are involved in MRSA’s peptidoglycan cell wall biosynthesis. Elsewhere, 7,9-Di-tert-butyl-1-oxaspiro (4,5) deca-6,9-diene-2,8-dione-rich plant extracts exerted a potent antimicrobial activity against a plethora of microbes [43].

## 5. Conclusions

The study investigated the antimicrobial and anticancer effects of both the methanol extract and the fractions from *Raphionacme hirsuta*, reportedly used in the treatment of a plethora of infections, including cancer and opportunistic infections associated with HIV-AIDS. The carbon tetrachloride fraction exhibited potential antimicrobial and anticancer activity compared to other extracts and the fractions. The activity was more pronounced against *Mycoplasma hominis*. Furthermore, the fraction exerted a remarkable anticancer activity against MCF-7, HeLa, and Caco-2 cell lines *in vitro*. Although the preliminary *in vitro* study may agree with the use of the plant species in treating the reported infections, there is a need to explore its mode of action further. Furthermore, it is necessary to explore its cytotoxicity against normal cell lines to ascertain its safety. The fractionation of the extract increased its biological activity. The bioactive fraction contained compounds that includes 7,9-Di-tert-butyl-1-oxaspiro (4,5) deca-6,9-diene-2,8-dione, and Hexanedial, which have been reported to possess remarkable and noteworthy antimicrobial and anticancer activity.

## Figures and Tables

**Table 1:** Antimicrobial activity (MIC in mg/mL) of extract, fractions, and isolated compounds from *Raphionacme hirsuta*.

Plant Materials	Selected Microorganisms							
	*C.* *albicans*	*C.* *neoformans*	*K.* *pneumoniae*	*M.* *catarrhalis*	*E.* *coli*	*M.* *hominis*	*S.* *aureus*	*B.* *cereus*
**Extract**								
Methanol	6.25	0.31	3.13	≥12.5	≥12.5	**0.08**	0.16	0.16
**Fractions**								
Aqueous	6.25	6.25	0.16	0.16	0.16	0.31	0.31	0.31
Methanol–water	3.13	0.16	≥12.5	6.25	0.156	0.31	0.31	0.31
Carbon tetrachloride	0.16	**0.02**	0.16	**0.04**	**0.02**	**0.02**	**0.04**	0.63
Chloroform	≥12.5	0.31	0.31	0.16	**0.08**	**0.08**	0.31	0.31
*n*-hexane	0.31	**0.02**	0.31	0.31	0.31	**0.08**	0.16	0.31
*n*-butanol	1.25	0.31	0.31	≥12.5	**0.08**	**0.04**	0.31	1.25
**Control drugs**								
Amphotericin	0.013	0.001	-	-	-	-	-	-
Streptomycin	-	-	0.003	0.003	0.025	0.006	0.013	0.003
Vancomycin	-	-	0.025	0.013	0.100	0.013	0.200	0.100

**Table 2:** Cytotoxicity of (IC_50_ in µg/mL) *Raphionacme hirsuta* methanol extracts and its fractions.

Plant Materials	HeLa	MCF7-21	Caco-2
Extract	540.6 ± 0.89	>1000	>1000
Aqueous	214.87 ± 2.11	112.22 ± 0.28	95.77 ± 0.11
Methanol–water	212.26 ± 1.04	86.44 ± 0.722	156.28 ± 0.08
Carbon tetrachloride	**18.21 ± 0.001**	**25.22 ± 0.002**	**26.33 ± 0.004**
Chloroform	68.44 ± 0.22	88.23 ± 0.002	44.28 ± 0.002
*n*-hexane	145.33 ± 3.33	260.24 ± 2.35	>1000
*n*-butanol	88.22 ± 0.44	325.38 ± 0.08	>1000
Doxorubicin	2.88 ± 0.002	1.46 ± 0.001	2.55 ± 0.001

**Table 3:** Tentative compounds identified from analysis of the Carbon tetrachloride fraction from *Raphionacme hirsuta* bulb using GC-TOF-MS.

R.T. (min)	Tentative Name	Observed Ion *m*/*z*	Area %	Similarity	Formula
2.38478	Conessine	265.47	0.162	706	C_24_H_40_N2
**2.51654**	**Hexanedial**	**117.06**	**3.691**	**677**	**C_6_H_10_O_2_**
**2.54277**	**Di-n-decylsulfone**	**282.14**	**3.201**	**546**	**C_20_H_42_O_2_S**
2.86167	3-Penten-2-one, (E)-	84.08	0.230	899	C_5_H_8_O
3.37973	Methylcarbamothioic acid, O-butyl ester	163.04	1.586	588	C_6_H_13_NOS
3.54183	2-Hydroxy-2-methylhept-6-en-3-one	14.08	0.315	695	C_8_H_14_O_2_
3.72415	Tetrahydrofuran	72.09	0.350	704	C_4_H_8_O
4.69345	Hexanal, 2-ethyl-	106.11	0.195	766	C_8_H_16_O
5.30413	2-Pentanone, 4-hydroxy-4-methyl-	117.07	0.235	740	C_6_H_12_O_2_
6.56207	Phenol	94.07	0.396	856	C_6_H_6_O
6.80004	Undecane, 4,6-dimethyl-	143.22	0.298	808	C_13_H_28_
8.74722	1-Dodecene	168.23	1.022	948	C_12_H_24_
9.02751	Benzaldehyde, 2,5-dimethyl-	134.11	0.371	911	C_9_H_10_O
10.3975	Dodecanal	184.23	0.413	945	C_12_H_24_O
10.6661	Cyclohexane, 1,3,5-trimethyl-2-octadecyl-	268.11	0.352	727	C_27_H_54_
11.3601	Tetradecane	198.29	0.294	860	C_14_H_30_
11.4284	2-Ethylbenzothiol, S-trimethylacetyl-	222.22	0.408	721	C_13_H_18_OS
11.4585	3-Pentanone, 2,2,4,4-tetramethyl-	138.15	0.214	665	C_9_H_18_O
**12.1984**	**4-(4-tert-Butylphenyl)-1,3-thiazol-2-ylamine**	**232.25**	**3.329**	**741**	**C_13_H_16_N_2_S**
12.2784	(4aR,5S)-1-Hydroxy-4a,5-dimethyl-3-(propan-2-ylidene)-4,4a,5,6-tetrahydronaphthalen-2(3H)-one	232.25	0.178	746	C_15_H_20_O_2_
12.3761	1-Pentene, 2,4,4-trimethyl-	112.16	0.155	783	C_8_H_16_
12.8212	Dodecyl acrylate	240.23	0.992	931	C_15_H_28_O_2_
12.9336	Heptacosane	304.27	1.005	873	C_27_H_56_
12.9719	Tetracosane	282.41	0.172	853	C_24_H_50_
13.2213	Neopentane	72.14	0.200	733	C_5_H_12_
13.8248	Phenanthrene, 2-dodecyl-9,10-dihydro-	280.00	0.168	665	C_26_H_36_
13.8475	6-Hydroxy-4,4,7a-trimethyl-5,6,7,7a-tetrahydrobenzofuran-2(4H)-one	196.16	0.204	890	C_11_H_16_O_3_
13.9666	1-Nonadecene	277.10	0.167	891	C_26_H_36_
14.008	1,4-Benzenediol, 2,6-bis(1,1-dimethylethyl)-	222.22	0.249	865	C_14_H_22_O_2_
14.0502	Octadecane	254.37	0.262	917	C_18_H_38_
14.5644	2-Pentadecanone, 6,10,14-trimethyl-	268.35	0.714	930	C_18_H_36_O
14.8228	1,2-Benzenedicarboxylic acid, bis(2-methylpropyl) ester	263.21	0.463	934	C_16_H_22_O_4_
15.1824	4-Tetradecylmorpholine	257.35	1.439	882	C_18_H_37_NO
**15.4743**	**7,9-Di-tert-butyl-1-oxaspiro (4,5) deca-6,9-diene-2,8-dione**	**276.25**	**5.322**	**926**	**C_17_H_24_O_3_**
15.4846	Heneicosane	296.41	1.065	637	C_21_H_44_
16.0775	Hexadecane, 2-methyl-	239.44	0.423	681	C_17_H_36_
16.1051	*n*-Hexadecanoic acid	256.29	0.514	819	C_16_H_32_O_2_
16.1473	Dodecane, 2,6,10-trimethyl-	255.33	0.181	786	C_15_H_32_
17.6776	Alanine, N-methyl-N-methoxycarbonyl-, undecyl ester	270.30	0.295	859	C_17_H_33_NO_4_
18.661	2,8,9-Trioxa-5-aza-1-silabicyclo [3.3.3] undecane, 1-ethyl-	203.20	0.944	762	C_8_H_17_NO_3_Si
20.0137	3-Amino-2-hydroxyheptanoic acid, N, N, O-trimethyl-, methyl ester	212.27	0.315	699	C_11_H_23_NO_3_
21.32	1-Cyclohexyldimethylsilyloxy-3,5-dimethylbenzene	262.15	1.117	654	C_16_H_26_OSi
21.3332	Triacontane	309.90	0.204	803	C30H62
21.4871	4,8,12,16-Tetramethylheptadecan-4-olide	309.40	0.217	921	C_21_H_40_O_2_
22.6064	1H-Indene, 1-hexadecyl-2,3-dihydro-	310.73	2.425	654	C_25_H_42_
22.9161	2-Methylpentacosane	309.42	0.156	861	C_26_H_54_
24.1608	Butyldiundecylamine	268.65	0.183	812	C_26_H_55_N

## Data Availability

The data used in the current work is held by the authors and is available on request.

## Supplementary Materials

The following supporting information can be downloaded at: https://www.mdpi.com/article/10.3390/life16071154/s1, Figure S1: Chromatogram, mS data and major compounds identified from Carbon tetrachloride fraction.

## Abbreviations

The following abbreviations are used in this manuscript:

ATCC	American Type Culture Collection
BEA	BEA Benzene: Ethanol: Ammonium hydroxide
Caco-2	human colorectal carcinoma cells
CEF	Chloroform: Ethyl acetate: Formic acid
DMSO	Dimethyl sulfoxide
EMW	Ethyl acetate: Methanol: Water
GC-TOF-MS	Gas chromatography mass-spectrometry
HeLa	Human cervical cancer cells
HIV-AIDS	Human Immunodeficiency Virus
IC_50_	A concentration that inhibits the growth of cells
INT	*p*-iodo-nitro-tetrazolium chloride
MCF-7	Human breast adenocarcinoma cells
MIC	Minimum Inhibitory Concentration
MTT	Tetrazolium-based colorimetric
TB	Tuberculosis
TLC	Thin-layer chromatography
R_f_	Retardation factor.
