# Supplementary material for: In Vitro Antimicrobial and Cytotoxic Effects of Solvent-Fractionated Extracts from Raphionacme hirsuta (E.Mey.) R.A.Dyer (Apocynaceae) Bulbs
Source: Life (Basel). 2026 Jul 13;16(7):1154. doi: 10.3390/life16071154 (PMC13412674; doi:10.3390/life16071154)
Supplement: Supplementary file 1 [file life-16-01154-s001.zip › life-4112912-supplementary.pdf]

Peak True - sample "4", 7,9-Di-tert-butyl-1-oxaspiro(4,5)deca-6,9-diene-2,8-dione, at 15.4743 min, Area (Abundance)

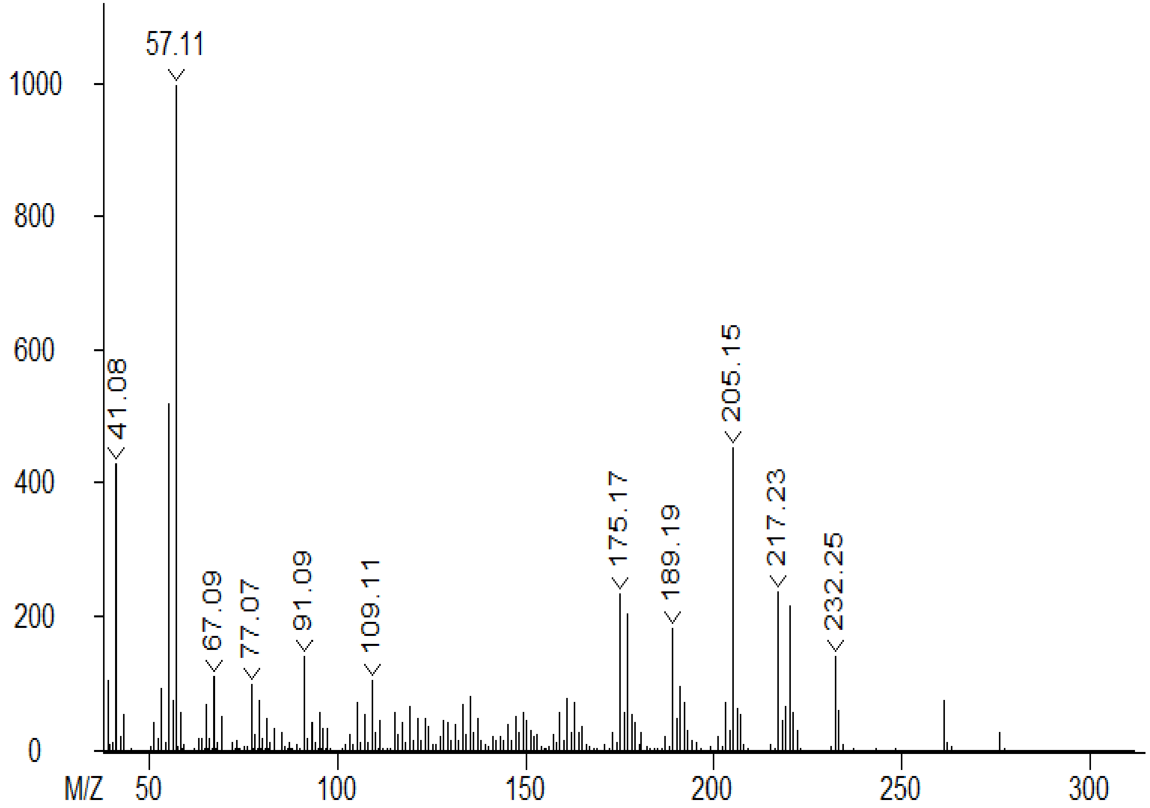

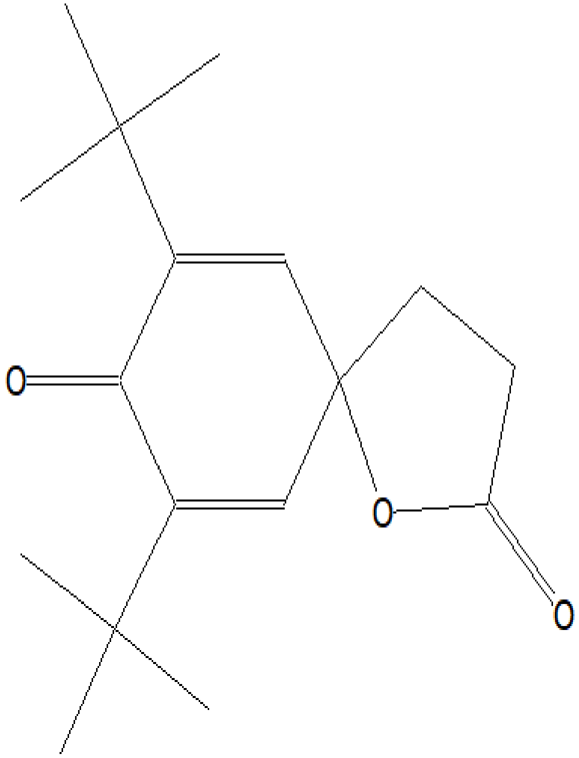

Peak True - sample "4", Di-n-decylsulfone, at 2.54277 min, Area (Abundance)

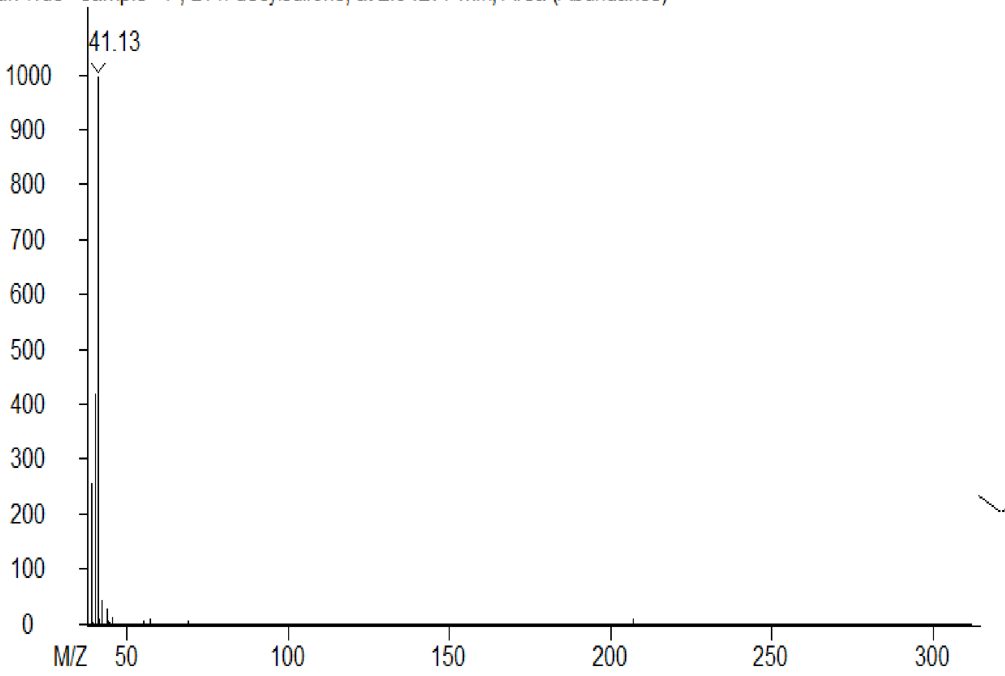

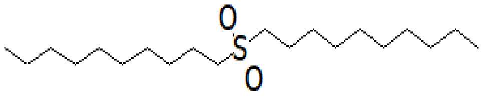

Peak True - sample "4", 4-(4-tert-Butylphenyl)-1,3-thiazol-2-ylamine, at 12.1984 min, Area (Abundance)

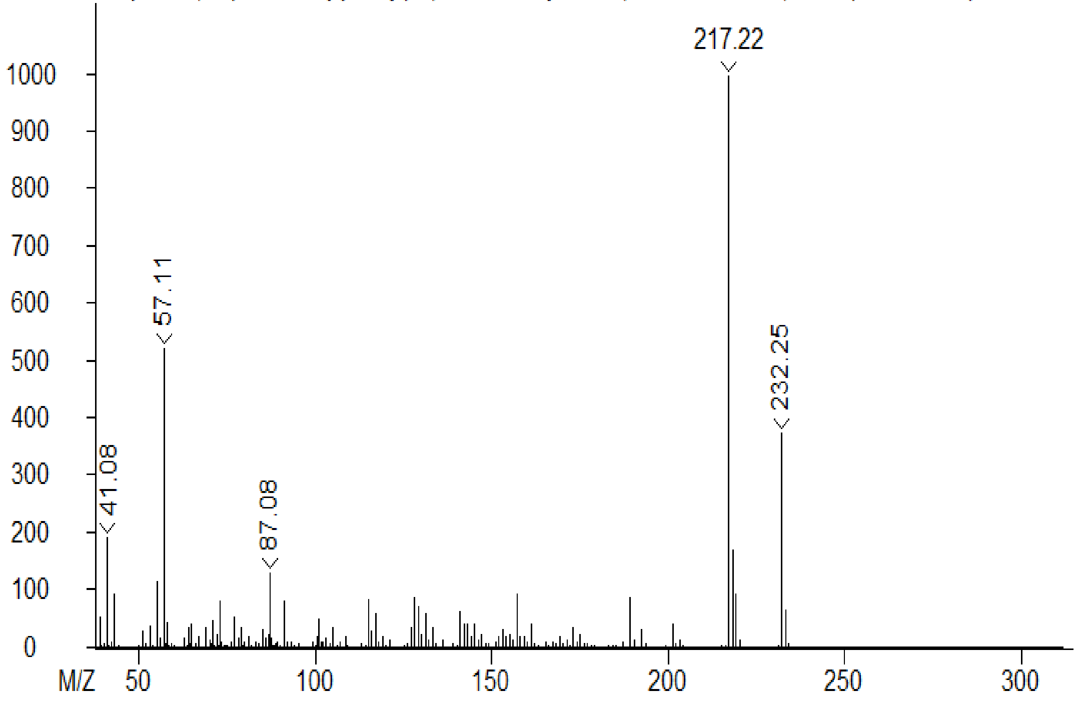

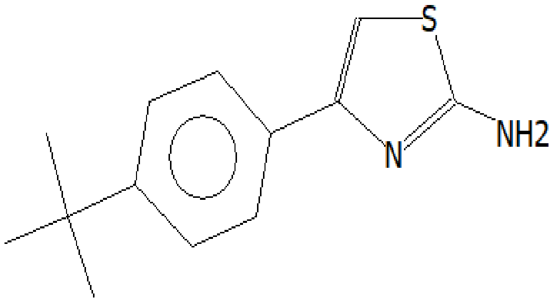

Peak True - sample "4", Hexanedial, at 2.51654 min, Area (Abundance)

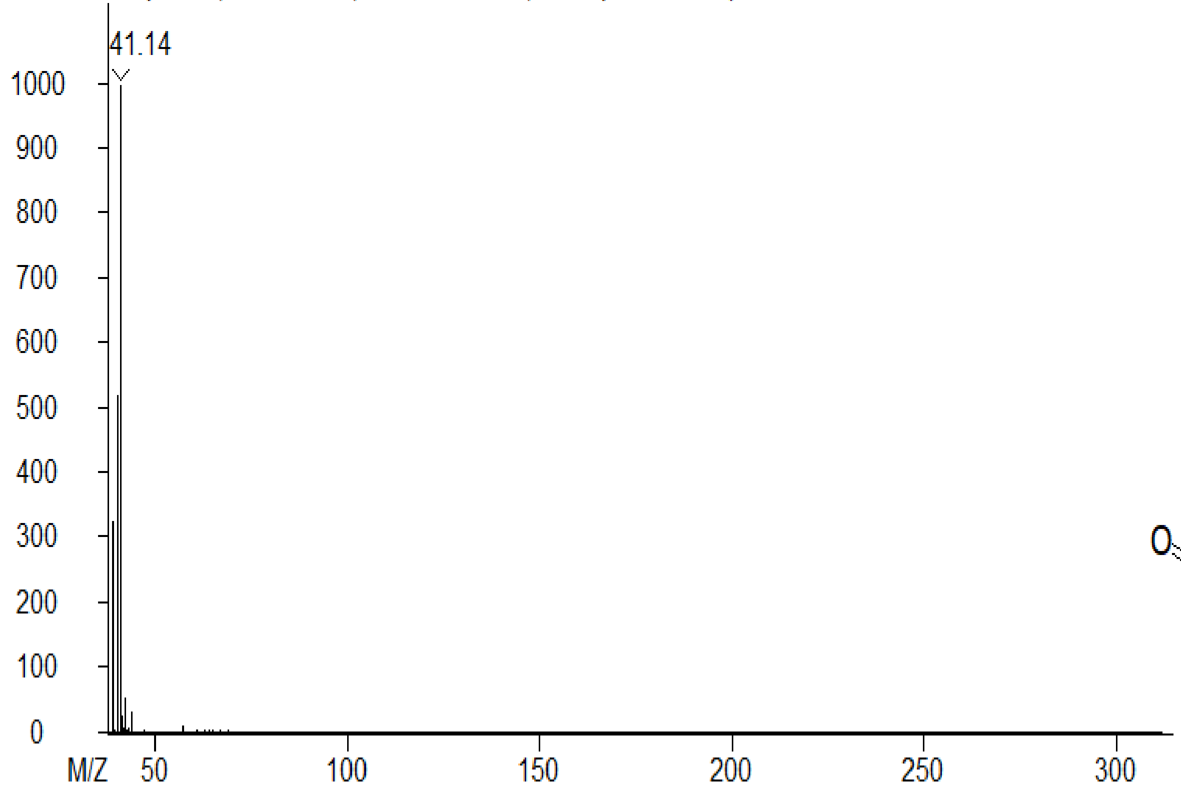

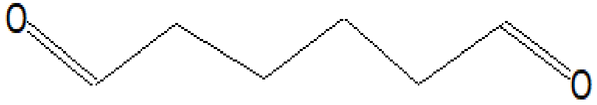

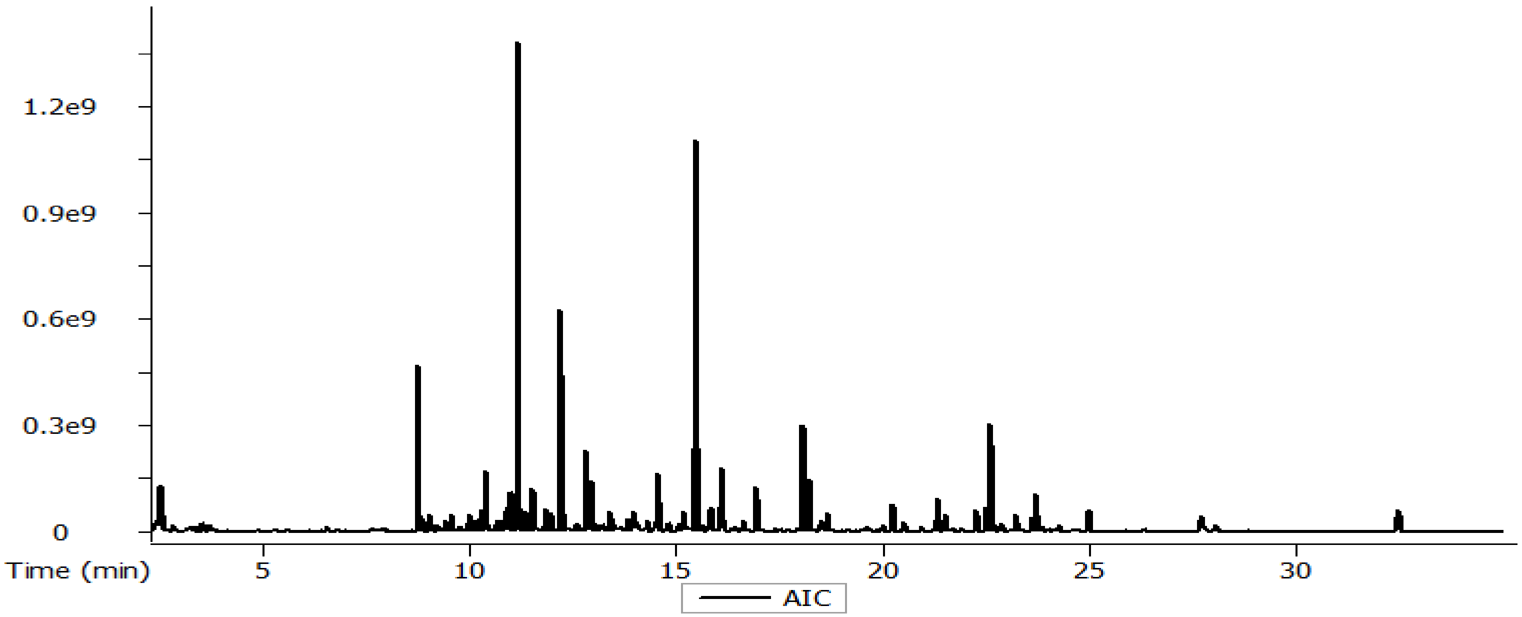

Chromatogram for the Carbon tetrachloride fraction
